# Bidirectional, non-necrotizing glomerular crescents are the critical pathology in X-linked Alport syndrome mouse model harboring nonsense mutation of human COL4A5

**DOI:** 10.1038/s41598-020-76068-4

**Published:** 2020-11-03

**Authors:** Jiang Ying Song, Nobuyuki Saga, Kunio Kawanishi, Kentaro Hashikami, Michiyasu Takeyama, Michio Nagata

**Affiliations:** 1 Kidney and Vascular Pathology, Faculty of Medicine, University of Tsukuba, 1-1-1, Ten-nodai, Tsukuba, Ibaraki 305-8577 Japan; 2 Axcelead Drug Discovery Partners, Fujisawa, Japan; 3 Department of Nephrology, Chongquing General Hospital, University of Chinese Academy of Sciences CGH, UCAS, Chongquing, China

**Keywords:** Chronic kidney disease, Glomerular diseases, Paediatric kidney disease

## Abstract

X-linked Alport syndrome (XLAS) is a progressive kidney disease caused by genetic abnormalities of *COL4A5.* Lack of collagen IV α5 chain staining and “basket-weave” by electron microscopy (EM) in glomerular basement membrane (GBM) are its typical pathology. However, the causal relationship between GBM defects and progressive nephropathy is unknown. We analyzed sequential pathology in a mouse model of XLAS harboring a human nonsense mutation of *COL4A5*. In mutant mice, nephropathy commenced from focal GBM irregularity by EM at 6 weeks of age, prior to exclusive crescents at 13 weeks of age. Low-vacuum scanning EM demonstrated substantial ragged features in GBM, and crescents were closely associated with fibrinoid exudate, despite lack of GBM break and podocyte depletion at 13 weeks of age. Crescents were derived from two sites by different cellular components. One was CD44 + cells, often with fibrinoid exudate in the urinary space, and the other was accumulation of α-SMA + cells in the thickened Bowman’s capsule. These changes finally coalesced, leading to global obliteration. In conclusion, vulnerability of glomerular and capsular barriers to the structural defect in collagen IV may cause non-necrotizing crescents via activation of PECs and migration of interstitial fibroblasts, promoting kidney disease in this model.

## Introduction

Alport syndrome (AS) is an inherited systemic disease caused by abnormalities of the gene encoding the collagen IV α-chain^1–4^. Kidney disease is a life-threatening organ involvement in AS that often leads to end-stage renal disease (ESRD)^5^. Collagen IV is widely distributed in the kidney and supports its structure and functions. In particular, the triple helix of collagen IV α3-α4-α5 chain is a core structure of the glomerular basement membrane (GBM)^6,7^, and genetic abnormalities in *COL4A3, COL4A4,* or *COL4A5* cause structural defects in GBM resulting in hematuria and proteinuria, which are the typical symptoms of AS.

AS is based on the three modes of inheritance. X-linked (XLAS) inheritance is the most common, and is caused by mutation of the *COL4A5* gene, which encodes the collagen IV α5 chain^2,5,8^. Other modes are autosomal dominant (AD) and autosomal recessive (AR) trait caused by mutations in the *COL4A3/COL4A4* genes^9,10^. Males with XLAS typically show early onset with severe phenotype leading to ESRD around 20 years of age, and thus, progressive kidney disease in XLAS is likely caused by structural abnormalities in GBM based on the genetic abnormality in *COL4A5*^2,5^.

Renal pathology in human males with XLAS has been described. Light microscopy (LM) reveals mesangial proliferation and focal segmental sclerosis in glomeruli, associated with fibrosis and tubular atrophy with frequent foam cell accumulation in the tubulointerstitial compartment^11,12^. However, these pathological features are not specific for XLAS and are found in the relatively advanced stage of the disease^13^. Therefore, it is unknown whether these LM features are caused by genetic abnormality of *COL4A5*. By contrast, lack of collagen IV α5 chain immunofluorescence (IF) in GBM and Bowman’s capsule has diagnostic value for XLAS. In addition, characteristic features as XLAS observed by electron microscopy (EM) called “lamination” or “basket weave” in GBM can be seen at a relatively early stage^14,15^. Although the lack of *COL4A5* in XLAS may cause negative IF result for collagen IV α5 chain and basket weave in GBM, and animal models of AS created by disruption of *Col4* genes uniformly showed abnormalities in GBM similar to human AS and glomerular sclerosis^16–19^, the linkage between “basket weave” and progressive kidney disease is not well characterized.

The present study focused on the sequential renal pathology in a recently established mouse model of XLAS carrying a nonsense mutation in exon 21 that is identical to one of the mutations found in human XLAS^20^.

Our findings indicate that non-necrotizing glomerular crescents are the culprit pathology leading to progressive kidney disease in our mouse model of XLAS. Pathogenesis of crescents seems to cause bidirectional damage to the glomerular and capsular barriers, both of which are physiologically pivotal to maintain the glomerular microenvironment depending on the normal function of *Col4a5*.

## Results

### Characteristic features in GBM in mutant mice

**Figure 1:**
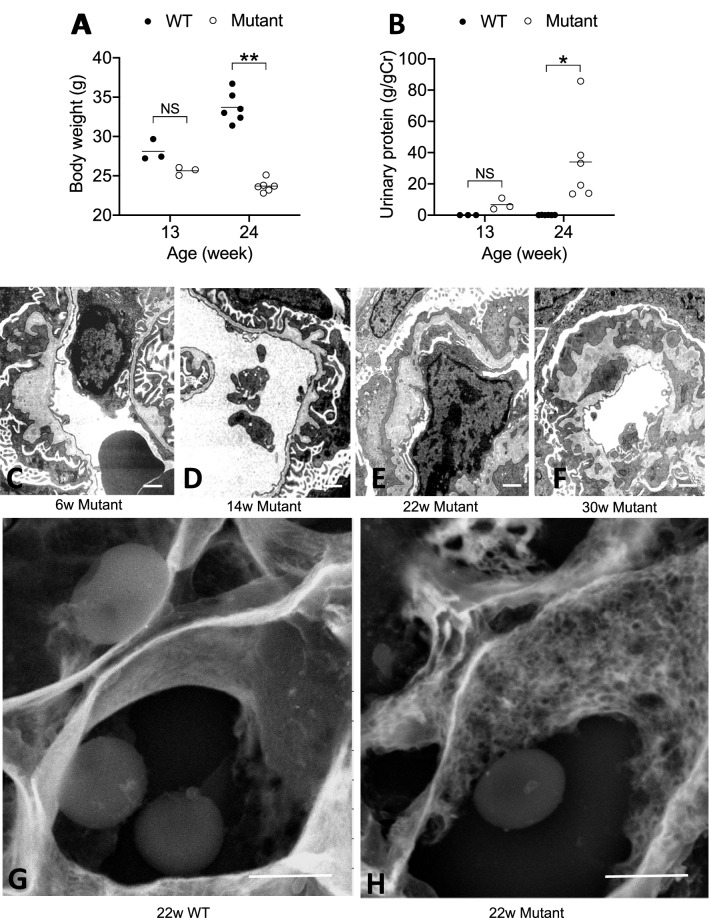
Characteristic ultrastructural changes of GBM in mutant mice. In mutant mice, body weight was significantly low (**A**) and proteinuria was significantly high (**B**) at 24 W compared to WT mice. Abnormality of GBM in mutant mice at 6 W showed focal out-pocket features (**C**). Thickening of GBM became accentuated and wide spread with increasing age: 14 W (**D**), 22 W (**E**), 30 W(**F**). Podocyte foot process effacement was associated with abnormal GBM and became diffuse with villous transformation. LVSEM revealed as ragged pattern of GBM in mutant mice at 22 W (**H**), whereas WT mice showed smooth surface (**G**). Scale bar 1.0 μm (**C**–**F**) and 2 μm in (**G**) and (**H**).

Mutant mice showed decrease of body weight and progressive proteinuria (Fig. 1A,B). Renal histological abnormalities in mutant mice first appeared as focal and segmental thickening with sparse matrices by TEM at 6 W. Note that foot process effacement in podocytes was closely associated with irregular GBM. GBM thickening became extreme and expanded with time, and mesangial matrices showed similar sparse features (Fig. 1C–F). 3D ultrastructure of GBM observed by low-vacuum scanning electron microscopy (LVSEM) revealed a ragged pattern of GBM with numerous pin-holes in mutant mice, in contrast to the smoothly arranged surface in WT mice (Fig. 1G,H). As previously reported, collagen IV a5 chain was entirely negative in the glomerulus and Bowman’s capsule^20^.

### Podocyte loss was not associated with the progression of kidney disease

**Figure 2:**
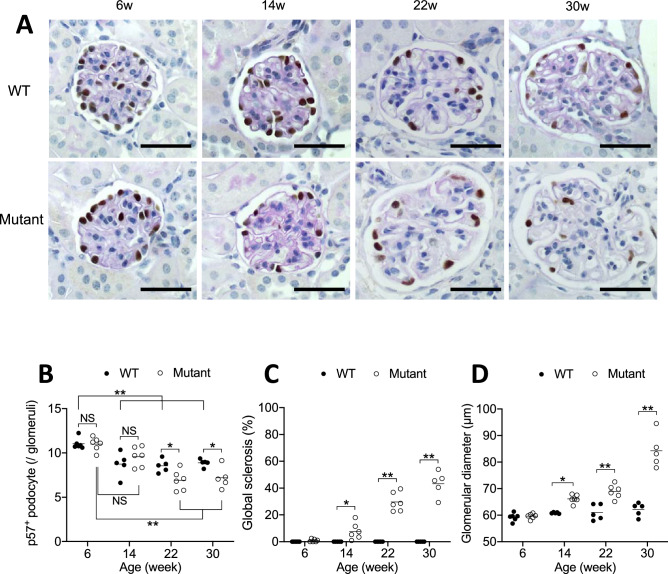
Preservation of podocyte number in unaffected glomeruli in the mutant mice until 14 W. Immunostaining with p57 with PAS staining in WT and mutant mice, shown are glomeruli without any injury (**A**). Note clear immunostaining of p57-positive cells limited to the podocyte. Average number of p57-positive cells per glomerular profiles showed no significant difference between WT mice and mutant mice in 6 and 14 week of age (**B**). Global glomerular sclerosis (**C**) was parallel with glomerular enlargement (**D**). Scale bar 20 μm in A.

**Figure 3:**
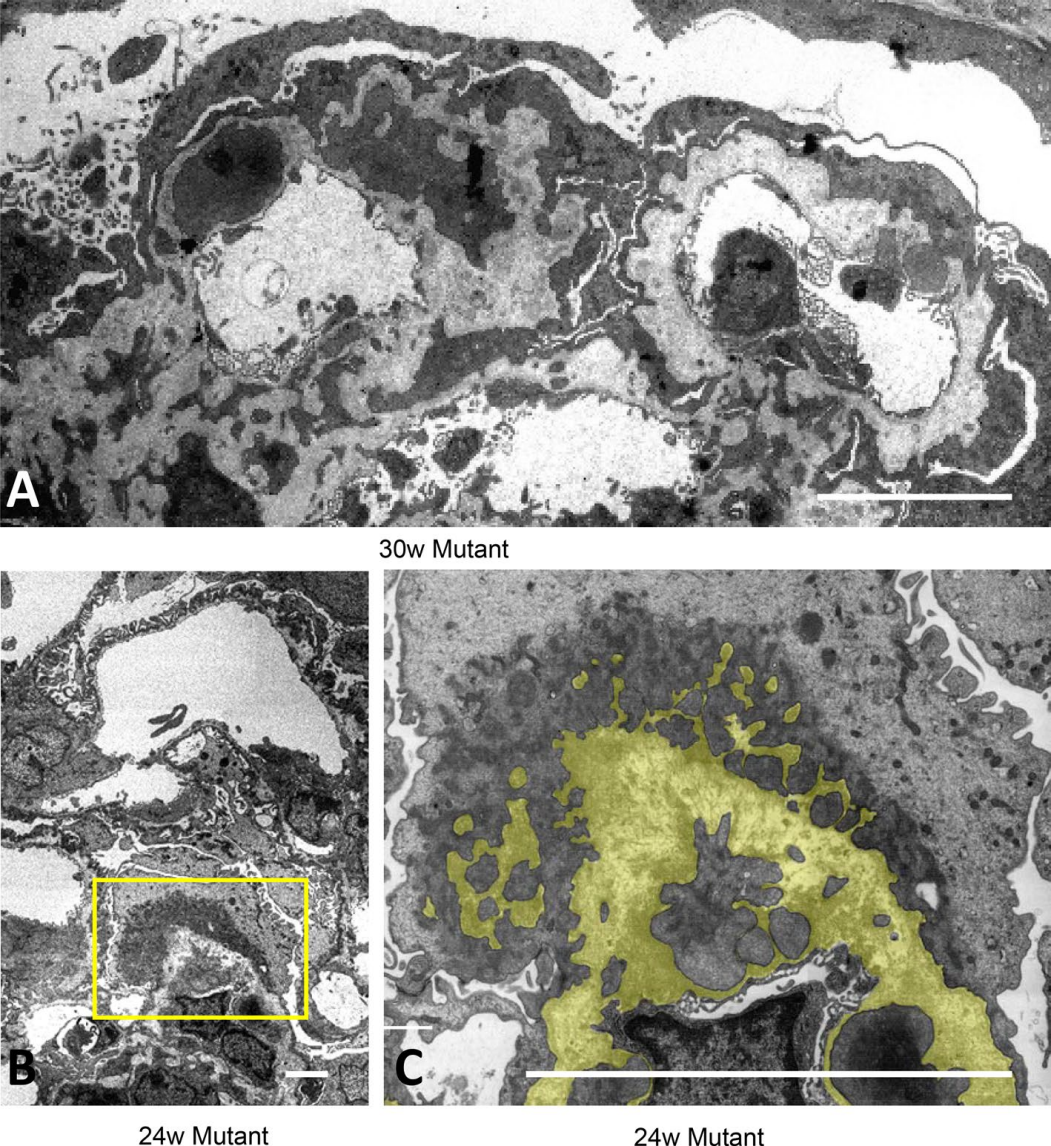
Diffuse podocyte effacement was associated with deep cell infolding into thickened GBM. TEM revealed extreme thickening of GBM and diffuse foot process effacement in podocytes in 30 W mutant mice (**A**). Severe foot process effacement with actin derangement in podocyte and cellular processes occasionally infolding into GBM in 24 W mutant mice (**B**, **C**). Yellow parts in (**C**) represents GBM. (**B**) is a larger magnification of the yellow square in (**B**). Scale bar, 5 μm (**A**–**C**).

Podocyte was revealed by p57 immunostaining (Fig. 2A). Average number of podocytes per glomerulus without abnormalities showed no change between WT mice and mutant mice until 14 W (Fig. 2B). At 22 W and 30 W, when a considerable number of glomeruli shows global sclerosis, remnant uninvolved glomeruli of mutant mice became significantly enlarged (Fig. 2C,D). TEM revealed substantially thickened GBM with sparse matrices as an out-pocket pattern (Fig. 3A). Podocytes showed diffuse foot process effacement with accumulation of actin, and remarkable podocyte infoldings into thickened GBM (Fig. 3B,C). Among 65 whole glomeruli inspected by TEM, podocytes did not show detachment despite severe changes in GBM and foot process effacement.

### Glomerular crescents were the exclusive glomerular lesion in mutant mice

**Figure 4:**
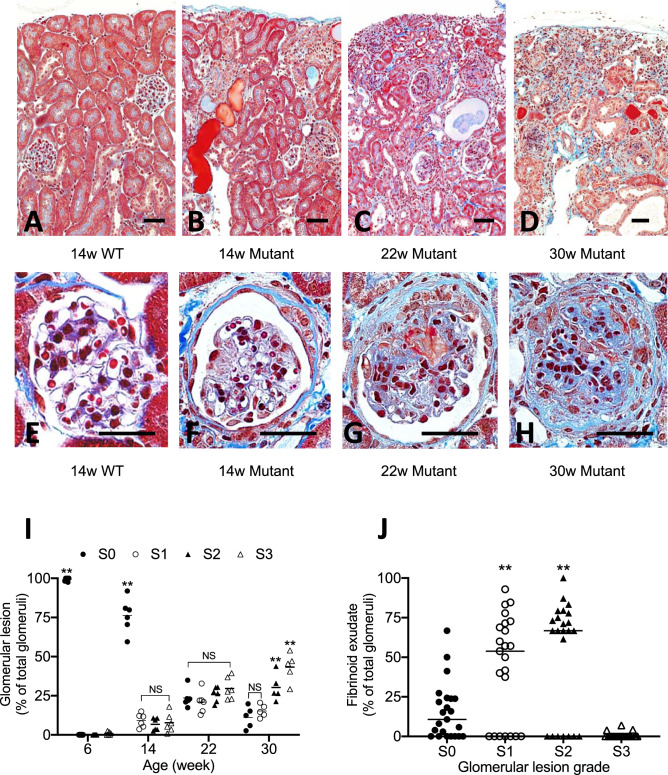
Glomerular crescent and associated tubulointerstitial damage were the cause of nephropathy in mutant mice. 14 W WT mice showed no structural abnormalities (**A**). Low magnification in mutant kidney showed glomerular abnormalities and associated tubulointerstitial damages at 14 W (**B**), 22 W (**C**) and 30 W of age (**D**). Thickening of Bowman’s capsule with cellular component (**F**) and cellular crescents with fibrinoid exudate (**G**) are two characteristic pattern of glomerular crescents in mutant mice, whereas the glomeruli appeared intact in WT mice (**E**). Globally obsolescent glomeruli often seen in 30 W mutant mice showed increased cellular component with matrices. Note that Bowman’s capsule was lost (**H**). Masson trichrome staining (**A**–**H**). Glomerular crescents were divided into 4 grades by the area occupied by crescents: S0 (no change), S1 (< 50%), S2 (> 50%), S3 (global sclerosis). In mutant mice, crescents were increase with time and S1, S2 were increased in 14 W and 22 W. Note that S0 and S1 were still presented at 30 W (**I**). Fibrinoid exudate was closely associated with S1 and S2, but not S3 (**J**). S0 glomeruli in 30 W mutant mice occasionally showed fibrinoid exudate. Scale bar, 20 μm (**A**–**H**).

WT mice did not show any crescents (Fig. 4A,E). Glomerular abnormality by LM in mutant mice was first noted at 14 W with uniform cellular crescents, which progressed at 22 W with extension of tubulointerstitial damage. At 30 W, mutant mice showed frequent glomerular obsolescence with more advanced tubulointerstitial damages (Fig. 4B–D).

Glomerular crescents revealed two patterns of formation. One was thickening of Bowman’s capsule with cellular component without glomerular abnormalities (capsular crescents, Fig. 4F). The other one was cellular crescents in the urinary space associated with fibrin-containing exudate identified by Masson trichrome stain (fibrin associated crescents, Fig. 4G). Loss of glomerular capsule was associated with cellular accumulation in the periglomerular region (Fig. 4H). By Masson trichrome stain, about 20% of glomeruli showed crescentic formation at 14 W, and the incidence and grade of crescents increased with time. Even in the advanced nephropathy of 22 W mutant mice, new cellular crescents (S1) were seen. In 30 W of mutant mice showed increase of global obliteration (S3), despite 20% of S0 or S1 were noted (Fig. 4I). Fibrinoid exudate was associated with crescents in S1 and S2 levels, but it was also found in glomeruli without crescents (S0) (Fig. 4J).

### Intracapillary fibrinoid exudate leaked into the urinary space and was involved in crescent formation

**Figure 5:**
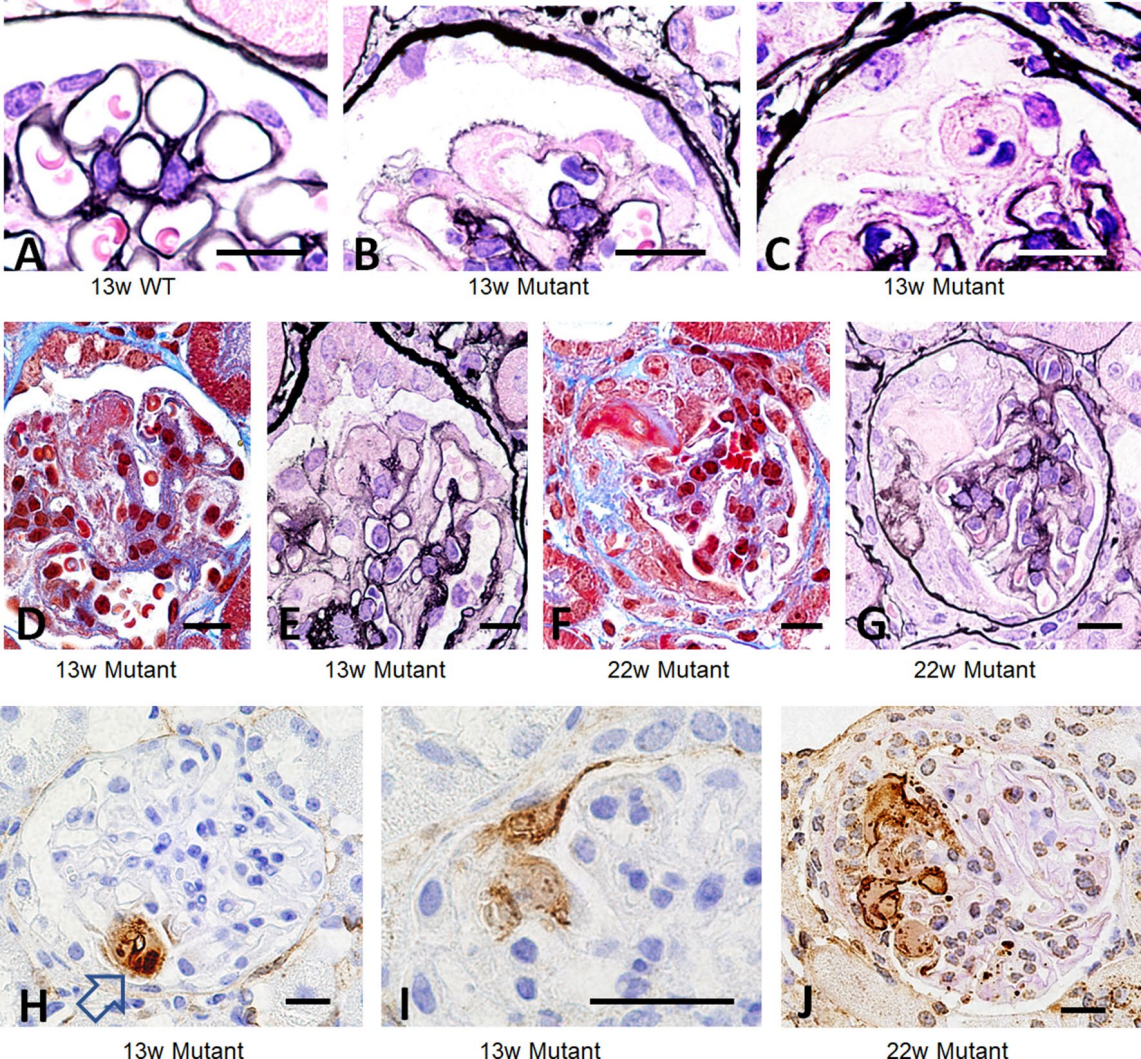
Intracapillary fibrinoid accumulate spilled into the urinary space, resulting in crescentic formation. WT mice at 13 W (**A**) showed clear outline of GBM stained by PAM as black line. Mutant mice at 13 W (**B**, **C**) revealed subendothelial/intracapillary accumulation of amorphous substances, which reduced PAM staining in GBM. These substances formed an exudate that spilled into the urinary space. Serial sections of Masson trichrome and PAM staining showed local fibrin thrombi (**D**, **E**) and fibrinoid exudate with loss of PAM staining in GBM (**F**, **G**) as revealed by serial sections. Immunohistochemistry with anti-fibrin antibody showed accumulation of fibrinoid exudate in the capillary (arrow) and spilt into the urinary space with crescentic formation (**H**–**J**). Scale bar, 10 μm (**A**–**J**).

**Figure 6:**
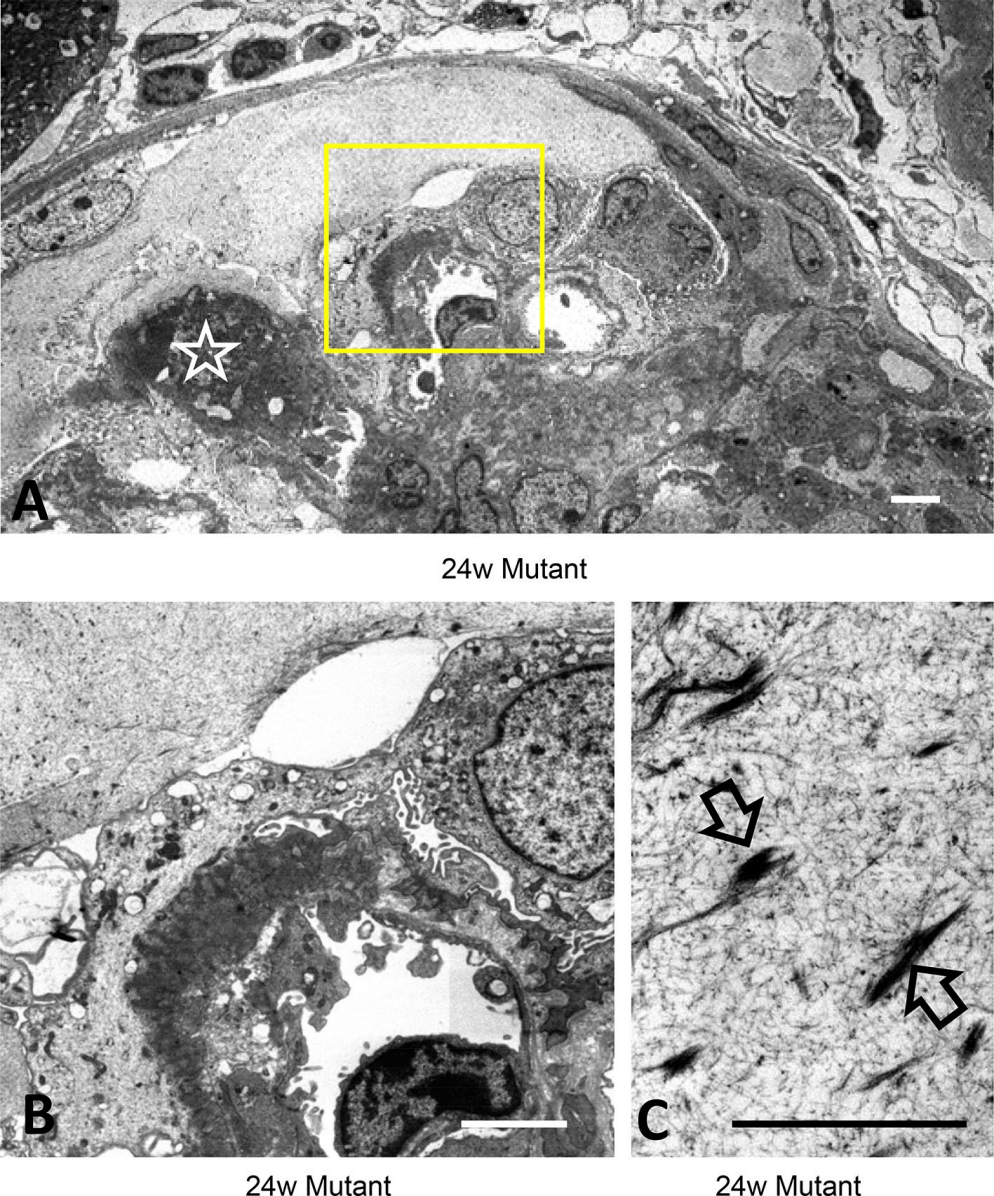
Focal loss of GBM was covered by podocytes and exudate contained sparse fibrin fibers. TEM at 24 W mutant mice revealed accumulation of clot in the capillary (white star) and local disappearance of osmiophilic demarcation of GBM staining (**B** is a larger magnification of yellow square in **A**). Note the fibrinoid structures sparsely in the exudate (**C**, yellow arrows). Scale bar, 5 μm (**A**, **B**) and 2.5 μm (**C**).

Early lesion of glomerular tuft in mutant mice showed segmental loss of PAM staining in GBM with intra- and extra- capillary amorphous exudate (Fig. 5A–C), whereas these features were entirely absent in WT mice. Serial sections with Masson trichrome stain and PAM stain showed that red fibrinoid deposition in capillary lumen and urinary space without showing GBM discontinuity (Fig. 5D–G). Immunostaining using anti-fibrin antibody showed focal accumulation of fibrin in the capillary, which leaked into the urinary space, and crescents developed with fibrin deposition (Fig. 5H–J). Lower-magnification views by TEM showed intracapillary fibrinoid deposition and focal fuzzy GBM, both of which were covered by podocytes (Fig. 6A,B). Higher-magnification showed that the fluid-like substances filling the urinary space contained fibrin fragments sparsely on a background of thin, fibrous material (Fig. 6C). The amorphous substance observed by PAM and the diluted fluid-like substance containing fibrin fragments seen by TEM were likely fibrin, as revealed by immunohistochemistry (Fig. 5H–J).

### Two types of crescents were composed either of CD44 or α-SMA-positive cells, but not macrophages

**Figure 7:**
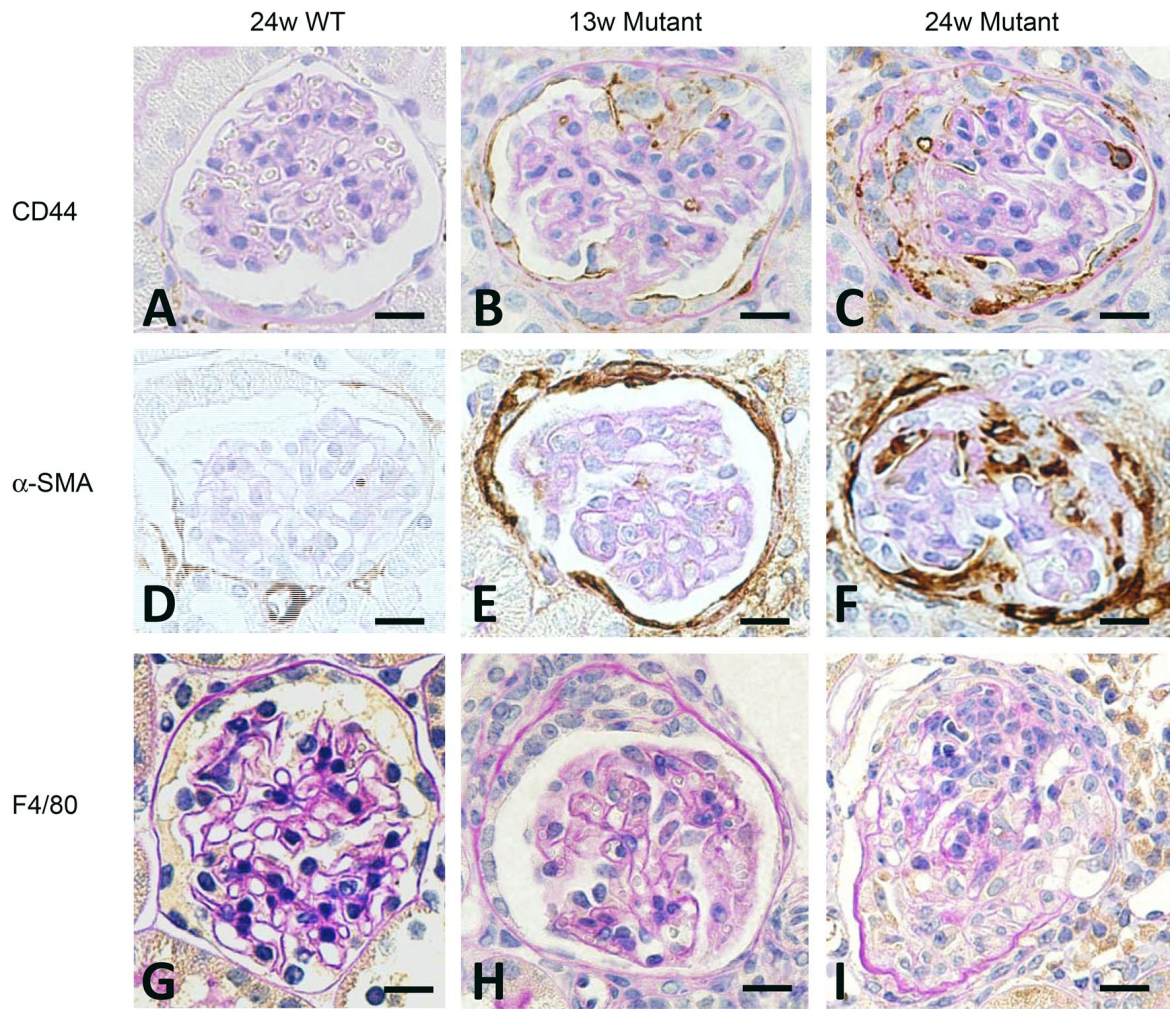
CD44 and α-SMA-positive cells were the distinct cellular component of crescents. In the WT mice, crescents were never observed, and intact glomeruli did not express CD44, α-SMA or F4/80 (**A**, **D**, **G**). Cellular crescents in 13 W mutant mice were composed of CD44 + cells which were increased with extension of crescent in the 24 W (**B**, **C**). Alpha-SMA + cells initially appeared within the thickened Bowman’s capsule (**E**) and migrated into glomerulus (**F**). Macrophages were entirely negative in crescent (**H**, **I**). Scale bar, 10 μm (**A**–**I**).

**Figure 8:**
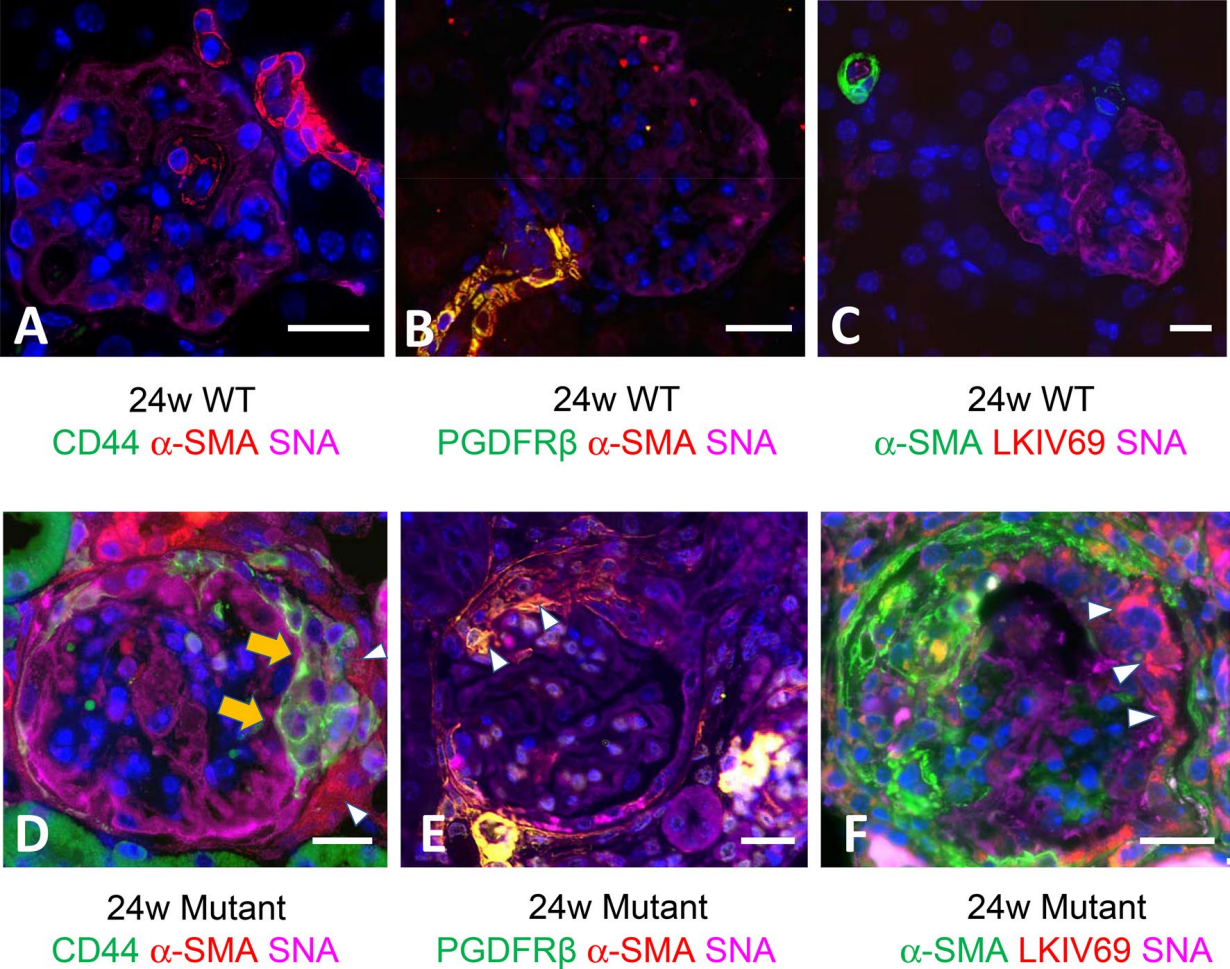
Expression of cell and extracellular matrices markers in the advanced crescents in 24 W by triple immunofluorescence in the mutant mice. Triple immunofluorescence with SNA (representing GBM) and α-SMA, and either CD44, PDGFR-β or LKIV69 in WT revealed that α-SMA and PDGFR-β were limited to the arteriolar wall. CD44 and LKIV69 were negative (**A**–**C**). The glomerulus with co-localization of fibrin-associated crescent (arrow) and capsular crescents (arrowhead) showed that the former was composed of CD44+ and the latter was α-SMA+ . Note double positive cells were absent. (**D**). Capsular crescent was α-SMA + and occasional co-expression of PDGFR-β (arrowhead) (**E**). In advanced crescent, α-SMA + cells are not associated with parietal cell matrices marker LKIV69 (arrowhead) (**F**). Scale bar, 10 μm (**A**–**F**).

**Figure 9:**
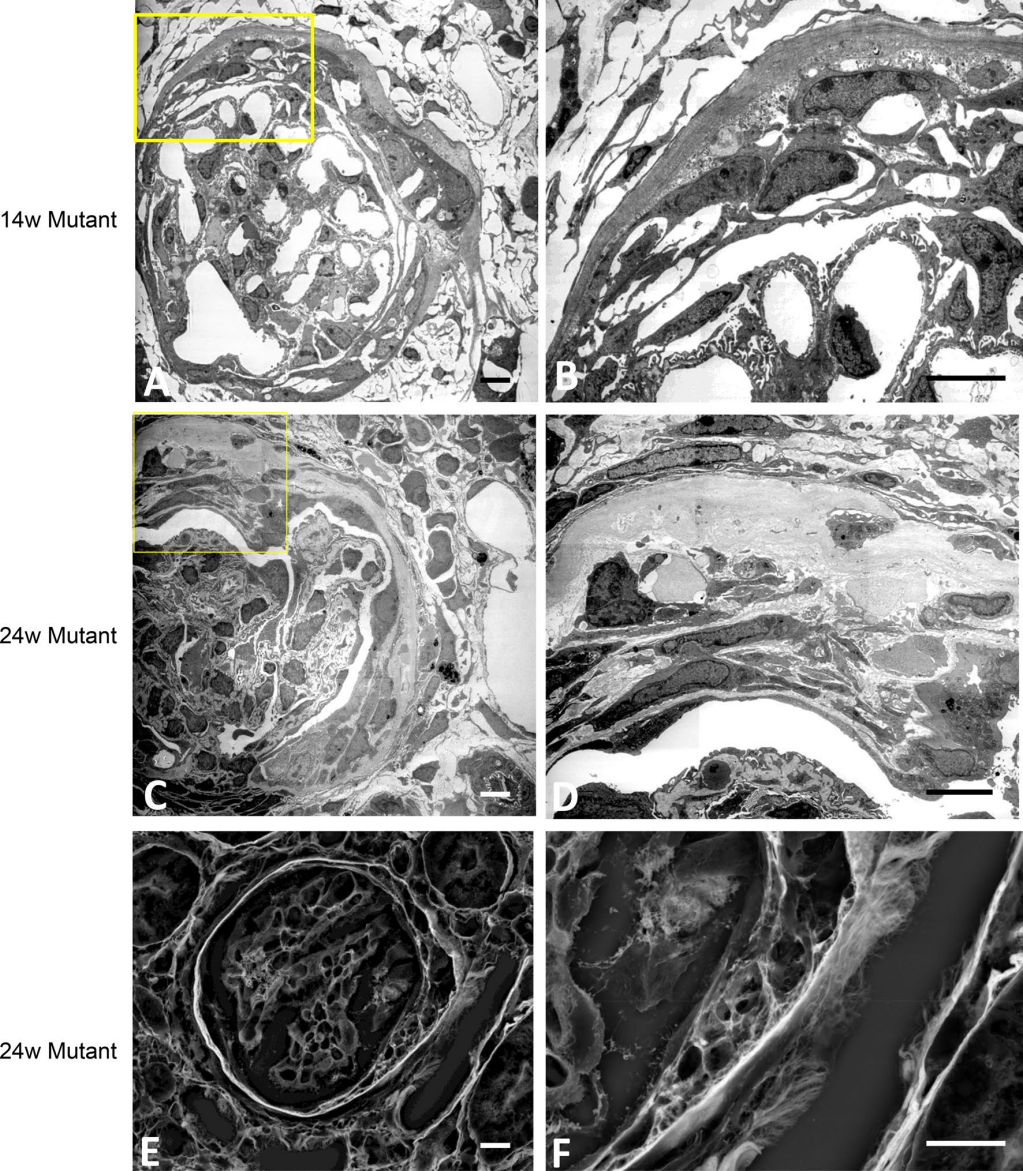
Accumulation of spindle-shaped cells within the thickened Bowman’s capsule. Early glomerular lesion showing thickening of Bowman’s capsule with spindle cells and extracellular matrices in 14 W mutant mice (**A**). Note that GBM and podocyte foot processes were nearly preserved (**B**). In the advanced lesion in 22 W mutant mice, Bowman’s capsule showed large splitting containing extra cellular matrices and spindle cells (**C**, **D**). The lining of PECs beneath the capsule was preserved. Splitting in Bowman’s capsule is well visualized by LVSEM as multiple lamellar structures reflecting disruption of collagen (**E**, **F**). Scale bar, 5 μm (**A**–**F**).

WT mice did not show crescents and immunohistochemistry showed that CD44 + cells, α-SMA + cells and F4/80 expressing macrophages were not present (Fig. 7A,D,G). In mutant mice, the fibrin-associated crescents revealed CD44 + epithelioid cells in the urinary space during progression (Fig. 7B,C). The capsular crescents without intraglomerular abnormalities was also noted in the early glomerular abnormalities, and its major component was α-SMA + cells, and CD44 and F4/80 + cells were absent (Fig. 7E,F,H). The advanced glomerular crescents were composed of CD44 + and α-SMA + cells, but not F4/80 + cells (Fig. 7C,F,I). Triple immunofluorescence using SNA and α-SMA, and either CD44, PDGFR-β or LKIV69 in WT mice revealed that only α-SMA and PDGFR-β were positive in the arteriolar wall, and both CD44 and LKIV69 were negative (Fig. 8 A–C). Fibrin-associated crescents were CD44 + but α-SMA-, whereas capsular crescents were composed of α-SMA + cells occasional co-localized with PDGFR-β, but CD44 was entirely negative. In case of association of two different crescents in one glomerulus, they were composed either of α-SMA + cells or of CD44 + cells and no double positive cells were seen. LKIV69 was not associated with crescent with α-SMA + cells (Fig. 8D–F). By TEM, capsular crescents showed accumulation of extracellular matrices and increased spindle-shaped cells in the split Bowman’s capsule (Fig. 9A–D). LVSEM revealed dissociation of Bowman’s capsule, whereas glomeruli showed no apparent abnormalities (Fig. 9E,F).

## Discussion

Progressive kidney disease in XLAS is caused by genetic abnormalities in *COL4A5*^4,8^. The present study employed a mouse model of XLAS carrying a nonsense mutation in *Col4a5* exon 21, one of the genetic abnormalities seen in human XLAS^20^, to investigate the linkage between mutation of *Col4a5* and progressive kidney disease.

In our model, segmental sparsely thickened GBM observed by TEM was the initial site of injury and appeared prior to the apparent changes in glomeruli and tubulointerstitial compartment. This is consistent with pathology in childhood XLAS showing that TEM-based GBM abnormalities are the earliest feature of pathology, followed by tubulointerstitial changes^11^. This indicates similar onset between our model and human XLAS.

The major finding of the present　study　was　that　cellular　crescents　were　the　exclusive　glomerular　pathology　promoting　progressive nephropathy in this model. The single nucleotide mutation in *Col4a5* caused exclusive crescents, and exon-skipping therapy enabled trimer formation of collagen IV and rescued glomeruli from crescents in this model^21^, indicating a causal relationship between this mutation and crescents.

Sequential analysis of kidney pathology yielded several key findings about the mechanism of crescent formation in this XLAS model. Glomerular crescents had two different origins: the major one was cellular crescents within the urinary space composed of CD44 + cells associated with fibrinoid exudate (fibrin-associated crescents). The other one was spindle cells accumulation in the thickened Bowman’s capsule (capsular crescents). This type of crescents was composed of α-SMA + -cells occasionally co-expressed PDGFR-β and not associated with parietal cell matrices LKIV69, suggesting that they were derived from periglomerular interstitial myofibroblasts. Different from necrotizing crescentic glomerulonephritis showing epithelial mesenchymal transition^22^, we found no double-positive cells for CD44 and α-SMA in this model.

Fibrin exudate was well correlated with active crescents (S1 and S2) and increased with time. Fresh cellular crescents were found even at a relatively late phase (22 W and 30 W), suggesting that local vulnerability of GBM and its resultant fibrinoid exudate was the basis of this type of crescents. Immunohistochemistry revealed that fibrinoid exudate was first seen locally in intracapillary space and leaked into the urinary space without disruption of GBM. This is consistent with the absence of hematuria during active crescentic formation in this model as reported^20^. Cellular crescents are generally caused by intra-capillary necrosis with GBM rupture^23^. However, negative IgG deposition in GBM (data not shown) excluded anti-GBM disease, and absence of macrophage infiltration and karyorrhexis, suggesting a non-necrotizing mechanism may underlie fibrin-associated crescents in this model. Based on the previous finding that fibrin or tissue factors stimulate crescent formation in anti-GBM disease^24,25^, we surmise that *Col4a5* mutation caused characteristic ragged GBM, as also seen in human AS^26^, permitted leakage of fibrinoid exudate, which may activate PECs to form crescents. These findings fully support the previous report showing that plasma leakage triggered non-inflammatory crescents in *Col4a3*-deficient Sv129 mice^27^.

In addition to GBM defects, vulnerability of Bowman’s capsule may be another plausible pathogenesis of crescents in this model. In the early stage of the kidney lesion at 14 W, mutant mice showed marked thickening of Bowman’s capsule associated with spindle and α-SMA + cell migration/proliferation with relatively preserved GBM and podocytes. The lesion occasionally grew having multilayered cells like crescents. Thickened Bowman’s capsule showed dissociation or splitting of Bowman’s capsular membrane filled with extracellular matrices by TEM and LVSEM. Alpha-SMA + cells occasionally coalesced with fibrin-associated crescents, leading to global obliteration with disruption of Bowman’s capsule. Diffuse glomerular crescents promoted tubulointerstitial damage, which is a hallmark of progressive nephropathy in variety of glomerular diseases. Our findings suggest that vulnerability of Bowman’s capsule promoted leakage of cells and exudate from the urinary space into the interstitial compartment, resulting in further extension of tubulointerstitial damage and subsequent progression. Although collagen IV α5 is a component of Bowman’s capsule and its defect may cause vulnerability of the capsular barrier, more studies are needed to understand the pathophysiology of Bowman’s capsule in progressive kidney disease in XLAS.

As shown in cellular/collapsing FSGS, non-necrotizing glomerular crescents composed of PEC hyperplasia are caused by podocyte loss^28–30^. By measuring podocyte number in renal biopsy samples from 21 patients with AS, a report suggested that podocyte depletion is the basis of progressive glomerulosclerosis in human AS^31^. This hypothesis seems to be reasonable, because abnormalities in GBM may disrupt the podocyte-GBM connection by integrin^32,33^, leading to podocyte detachment and subsequent segmental sclerosis. However, it is unknown whether podocytopenia is a primary trigger of sclerosis caused by dysregulation of podocytes or a secondary event associated with progressive nephropathy. In this regard, we showed that podocyte density in the uninvolved glomeruli was unchanged during progressive kidney damage until 14 weeks of age, indicating that podocyte depletion was not a forerunner of sclerosis in our model. Although podocyte foot process effacement was extensively observed in the glomeruli at an advanced stage, we did not observe podocyte detachment in any of injured glomeruli, including 59 whole glomeruli from mutant mice inspected by TEM. Instead, podocytes tended to invaginate their processes deeply into the thickened GBM, and are thus likely to resist detachment, as has been suggested in other models of podocyte injury^34,35^. Because injured podocyte synthesized chemokines which stimulated PEC activation via CXCR4^36^, podocyte injury in this model may also be involved in the crescentic formation, even without detachment. Thus, glomerular crescents produced by disruption of two functional barriers, but not FSGS caused by podocyte depletion, is the primary pathogenesis promoting kidney disease in our model.

Although the initial GBM changes observed by TEM in this model were similar to those in human XLAS, exclusive crescents with fibrinoid leakage were not typical of what we see in human biopsy samples, even though few unusual cases with crescents were reported^37,38^. The severer pathology in this model may result from the type and spot of mutation. Nonsense mutation of exon21 may cause substantial dysfunction of collagen IV α5 resulting high permeability in GBM and vulnerability of Bowman’s capsule, both of which synergistically promote progressive nephropathy via crescents. We surmise that pathology in our mice may represent an accentuated, but principle pattern of kidney injury due to serious dysfunction of collagen IV α5.

The present study has limitations. As has been discussed, the pathogenesis of crescents may require 3D morphology to be fully represented, whereas we observed crescents mostly by 2D. To avoid misinterpretation of the genesis of crescents based on 2D images, we observed all the glomeruli in one kidney section, about 50–60/animal, and total of 65 whole glomerular profiles by TEM. In addition, we used LVSEM to demonstrate 3D appearance of GBM, which impacted to represent high GBM permeability. Additional studies of crescent morphogenesis using modern techniques, such as cell lineage studies or multiphoton microscopy may provide further information for interpreting the pathogenesis of glomerular crescents in XLAS.

In conclusion, mutation of *Col4a5* caused disruption of GBM and Bowman’s capsule barriers, leading to bidirectional non-necrotizing crescents and subsequent tubulointerstitial damage. Our findings suggest that XLAS is a disease of the glomerular and capsular barriers, which maintain the glomerular microenvironment.

## Methods

### Mice

We employed a recently established mouse model of XLAS harboring a nonsense mutation (R471X) of *COL4A5* in human XLAS, using the clustered regularly interspaced short palindromic repeat (CRISPR)/Cas9^20^. All mice had ad libitum access to water and standard chow diet (CE-2; CLEA Japan) and were housed in a temperature- and humidity-controlled room. Animal experiments were approved by the Institutional Animal Care and Use Committee of the University of Tsukuba (Registration No.19052) in accordance with institutional guidelines. In total, 64 male mice were used. Forty-six kidney samples were obtained in the initial study: 6 W (WT n = 6, Mt n = 6), 14 W (WT n = 6, Mt n = 6), 22 W (WT n = 6, Mt n = 6), and 30 W (WT n = 5, Mt n = 5)^20^. Eighteen mice were added for further analysis: 13 W (WT n = 3, Mt n = 3) and 24 W (WT n = 6, Mt n = 6).

### Measurement of proteinuria

Urine samples were collected in metabolic cages for 16 h from the age of 13 W and 24 W weeks in an additional group of mice. Urinary albumin (Alb) level was measured using ELISA with Albuwell M (Exocell, Philadelphia, USA). Urinary creatinine (Cre) level was measured using a Hitachi 7170 type automatic analyzer (Hitachi, Tokyo, Japan) with L-Type Wako CREM (Wako Pure Chemicals Industries, Osaka, Japan). Urinary albumin levels were expressed as U-A1b/ U-Cre.

### Staining and morphological analysis

The kidney samples were used for either light microscopy (LM), immunofluorescence or transmission electron microscopy (TEM) by standard procedures. For LM, 4% paraformaldehyde-fixed tissue was sectioned and stained with hematoxylin and eosin (HE), Periodic acid Schiff (PAS), periodic acid methenamine silver (PAM) and Masson trichrome (MT). For immunohistochemical staining, we used antibodies against p57 (goat polyclonal, Santa Cruz Biotechnology, Santa Cruz, CA, × 200 dilution), CD44 (mouse monoclonal, BD Pharmingen, Franklin Lakes, NJ, × 50 dilution), α_SMA (rat monoclonal, Sigma-Aldrich USA, × 500), F4/80 (rat monoclonal, AbD Serotec, Oxford, UK × 100), and fibrin (rabbit polyclonal, Dako Cytomation Denmark × 1000). Antigen retrieval by citrate buffer (10 mM, pH 6.0) with microwave was done for CD44 and F4/80 staining. Secondary antibodies were EnVision + System-HRP Labeled Polymer anti-rabbit (Dako, Glostrup, Denmark) for a rabbit primary antibody, Biotin Conjugated Histofine (Nichirei Bioscience, Tokyo, Japan) for a mouse primary antibody, and Biotin goat anti-rat IgG (Cedarlane, Burlington, Canada, × 100) for a rat primary antibody. After the sections were reacted with Peroxidase Streptavidin, immune product was visualized by diaminobenzidine (DAB substrate-chromogen system, Dako). Triple immunofluorescent microscopy for SNA and α-SMA, LKIV69 (a kind gift from Dr. Smeets and Dr. van Kuppevelt), PDGFR-β (goat polyclonal, R&D systems USA × 100) or CD44 was performed with antigen-retrieved paraffin-embedded sections. Goat anti-rat IgG (H + L) Alexa 488 and anti-vsv-cy3 antibody were used as secondary antibodies, respectively. To identify GBM, Cy5 Sambucus Nigra (SNA) (Vector Laboratory, Inc, CA, USA, × 500) which labels GBM was also applied. IF images were captured with BZ-X800 (KEYENCE, Osaka, Japan). For 3D inspection, we used low-vacuum scanning electron microscopy (LVSEM) with PAM-stained 5 μm thick paraffin sections (Hitachi TM-1000 or TM3030; Hitachi Co. Ltd., Tokyo) without cover slips as described elsewhere^29^. LVSEM observations were made at an acceleration voltage of 15 kV with 30 Pa. TEM (Hitachi H-7100; Hitachi Co., Ltd., Tokyo) was processed by standard protocol and inspected at an acceleration voltage of 80 kV. In total, we inspected 65 whole glomeruli (WT 6, Mt 59) in total by TEM.

### Histopathological analysis

Podocyte density was calculated in each glomerulus as p57 + cell number/glomerular area. Glomerular planar area and glomerular diameter were calculated using a virtual slide system (NDP viewer, Hamamatsu Photonics). We measured all the glomeruli encountered in each section (on average, 93.1 glomeruli/animal). CD44-positive glomeruli were quantified by the incidence of glomeruli having CD44 + cells. Similar to p57, we analyzed CD44 + glomeruli among all the glomeruli encountered in each section (on average, 97.7 glomeruli/animal). To express the degree of glomerular injury in each animal, glomerular scoring was performed. Glomerular lesions were divided into four grades using PAM-stained slides depending on the glomerular area involved by crescent; S0 (no change), S1 (< 50%), S2 (> 50%), S3 (global sclerosis). All the glomeruli in the single section in each animal were individually scored, and the average value in each animal was calculated. On average, over 90 glomeruli per section were examined. Fibrinoid exudate was estimated by MT staining showing red-colored amorphous deposition in the tuft or urinary space. The incidence of fibrin-positive glomeruli per section was calculated.

### Statistical analysis

Data were analyzed by mixed-effects analysis with Sidak’s multiple comparisons test in Figs. 1A,B, 2B,C and 4I and by one-way analysis of variance (ANOVA) with Tukey’s multiple comparisons test in Fig. 4J. All results are presented as a scatter plot with mean. Statistical analyses were performed using Prism software (version 8; GraphPad Software) and statistical significance was indicated by *p < 0.05 and **p < 0.01 levels.

## Supplementary information

Supplementary information

## Data Availability

The datasets generated and analyzed during the current study are available from the corresponding author on reasonable request.
