# Supplementary material for: Bidirectional, non-necrotizing glomerular crescents are the critical pathology in X-linked Alport syndrome mouse model harboring nonsense mutation of human COL4A5
Source: Sci Rep. 2020 Nov 3;10:18891. doi: 10.1038/s41598-020-76068-4 (PMC7642296; doi:10.1038/s41598-020-76068-4)
Supplement: Supplementary file 1 — Supplementary information [file 41598_2020_76068_MOESM1_ESM.pptx]

## Slide 1
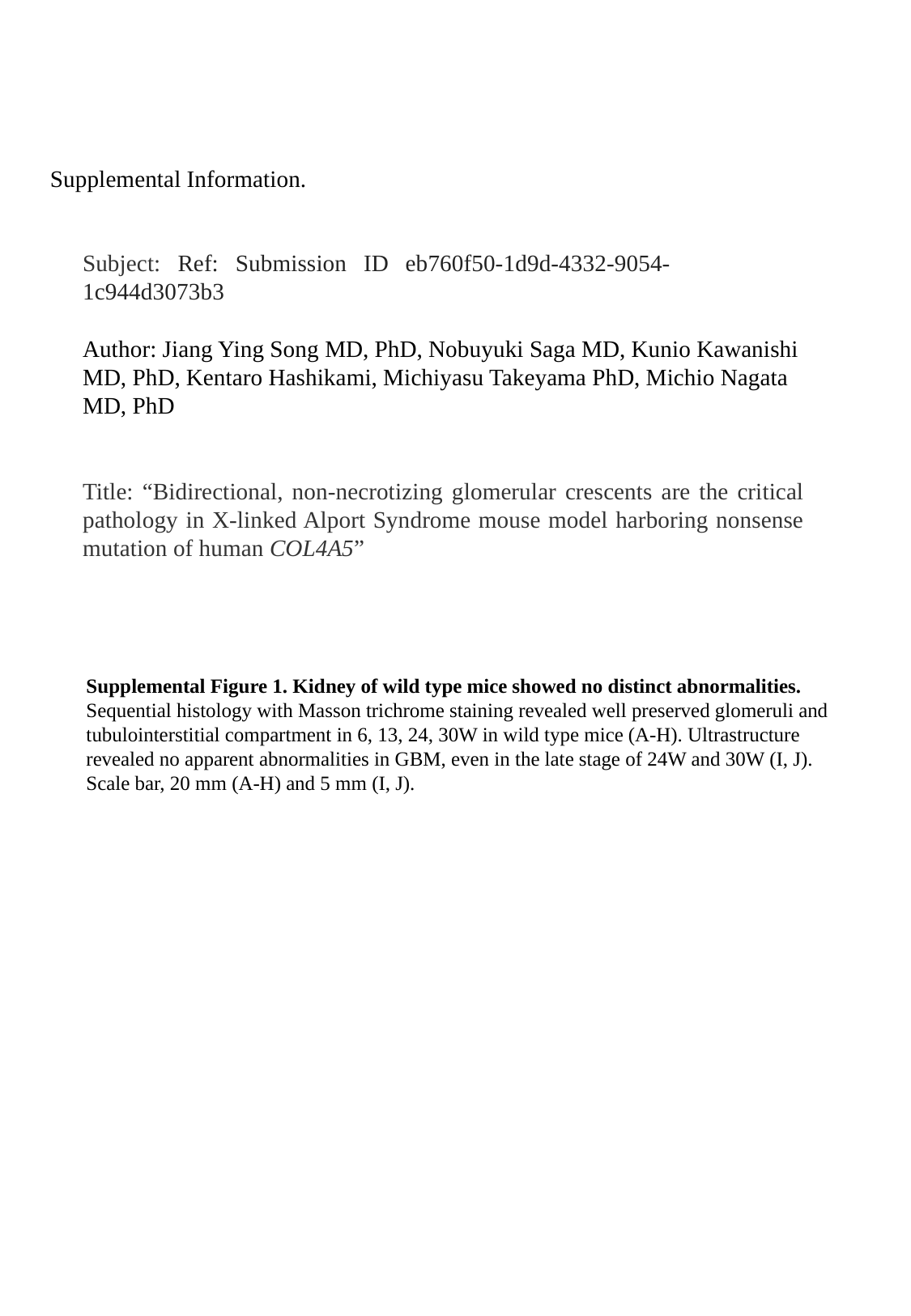

Supplemental Information.
Subject: Ref: Submission ID eb760f50-1d9d-4332-9054-1c944d3073b3
Author: Jiang Ying Song MD, PhD, Nobuyuki Saga MD, Kunio Kawanishi MD, PhD, Kentaro Hashikami, Michiyasu Takeyama PhD, Michio Nagata MD, PhD
Title: “Bidirectional, non-necrotizing glomerular crescents are the critical pathology in X-linked Alport Syndrome mouse model harboring nonsense mutation of human COL4A5”
Supplemental Figure 1. Kidney of wild type mice showed no distinct abnormalities. Sequential histology with Masson trichrome staining revealed well preserved glomeruli and tubulointerstitial compartment in 6, 13, 24, 30W in wild type mice (A-H). Ultrastructure revealed no apparent abnormalities in GBM, even in the late stage of 24W and 30W (I, J). Scale bar, 20 mm (A-H) and 5 mm (I, J).

## Slide 2
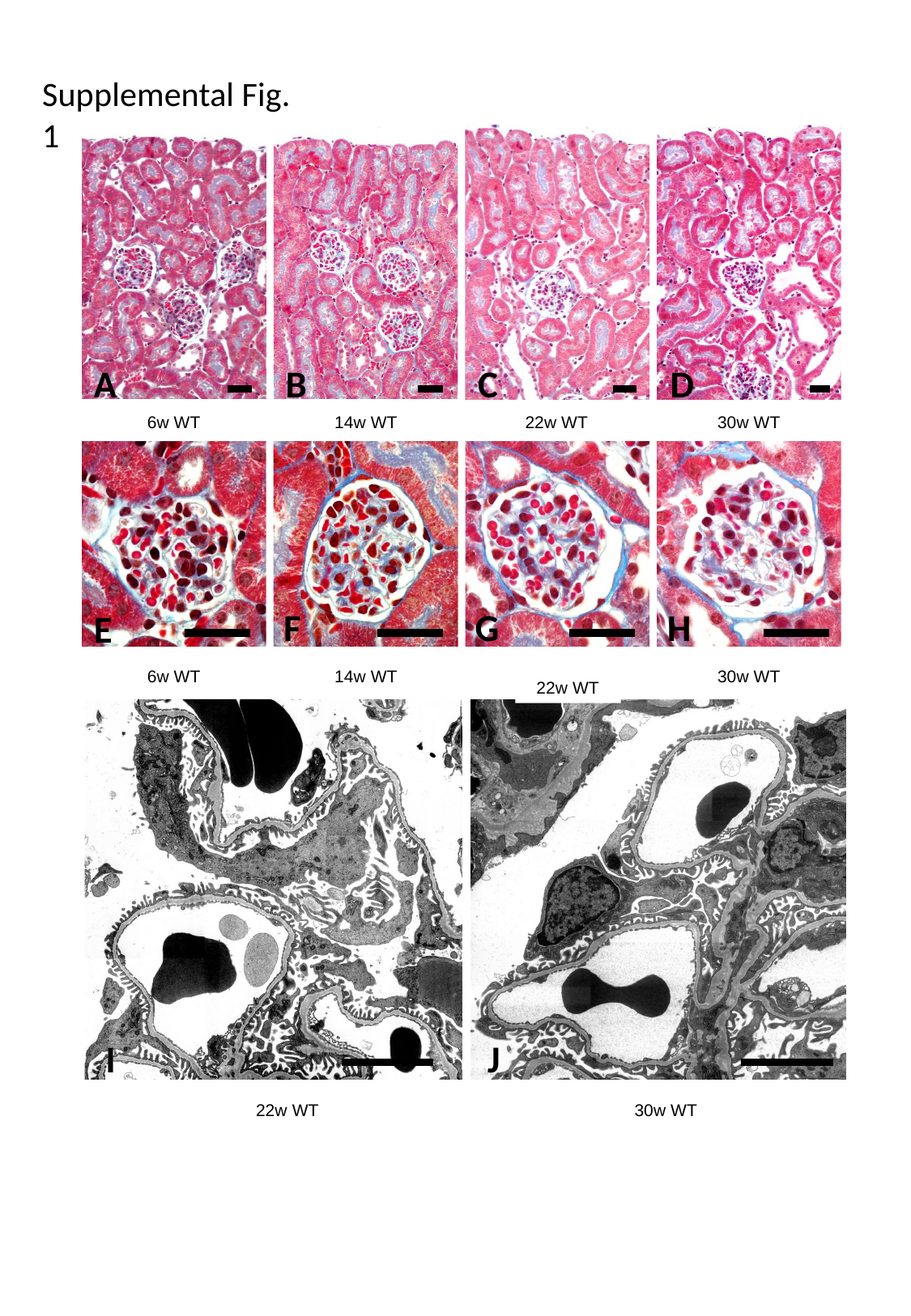

Supplemental Fig. 1
I
A
B
C
D
6w WT
14w WT
22w WT
30w WT
F
G
H
E
6w WT
14w WT
30w WT
22w WT
I
J
22w WT
30w WT
